# Co-creating public health measures with adolescents in municipalities: Municipal actors’ views on inhibitors and promoters for adolescent involvement

**DOI:** 10.1177/14034948231170430

**Published:** 2023-05-04

**Authors:** Mari Sylte, Monica Lillefjell, Kirsti S. Anthun

**Affiliations:** 1 Department of Neuromedicine and Movement Science, Norwegian University of Science and Technology, Norway; 2 Department of Public Health, University of Stavanger, Norway; 3 Department of Health Research, SINTEF Digital, Norway

**Keywords:** Health promotion, public health measures, adolescent, co-creation, community participation, qualitative research

## Abstract

**Aim:**

To explore what municipal actors consider as inhibiting and promoting adolescents’ involvement in public health measures in municipalities.

**Methods:**

A qualitative study with individual and group interviews was conducted among 15 municipal actors who were central in involving adolescents from five Norwegian municipalities participating in the National Programme for Public Health Work in Municipalities (2017–2027). In addition, participatory observation of project activities was done in two municipalities. A data-driven thematic analysis was applied to analyse data.

**Results:**

In the analysis, we developed four themes, including both inhibitors and promoters for adolescent involvement: (a) Timeframe challenges in adolescent involvement; (b) Lack of necessary knowledge and awareness among adolescents; (c) Limited competencies and resources in the project groups; and (d) Facilitators’ attitudes on and perceptions of adolescent involvement.

**Conclusions:**

**This study reports factors that are important to consider when facilitating involvement processes with young people. Findings suggest that further work should be done to ensure involvement of adolescents in public health measures in municipalities, and actors involving adolescents must be provided with competence and resources to ensure such participation.**

## Background

The involvement of citizens in community development is often viewed as crucial to solving many of our current and future health challenges. Complex problems and demands cannot be met solely by the public sector but rely on collaboration between public and private actors and citizens [1]. This is also reflected in the United Nations Sustainable Development Goals (SDGs), in which one goal is devoted to partnerships, emphasising that only through collaboration and cooperation can the SDGs be fulfilled [2]. The active involvement of citizens in community development represents a shift from viewing citizens as passive receivers to active, empowered, and resourceful contributors. Such processes, called co-creation, co-production, or co-designing [1], enable citizens and governments to work together towards mutual goals and can address the need for innovation and collaboration in health promotion [3].

Conversely, co-creation has been criticised for allowing authorities to disclaim responsibility by shifting it onto individual citizens, which can lead to unintended effects such as loss of democracy and distrust if the co-creators’ expectations are not met, and has been shown to increase social inequalities [4, 5]. An explanation for the latter is that people with higher socioeconomic status tend to participate more in co-creation processes, assert more influence, and benefit more from being engaged than less privileged citizens [5].

Health promotion is defined as the process of enabling individuals to gain control over and improve their health [6]. Hence, empowerment and inclusive co-creation processes are fundamental in reaching this. Hart [7] describes participation as ‘the process of sharing decisions which affect one’s life and the life of the community in which one lives’. The involvement of children and adolescents has received gradually heightened attention since 1989, when the United Nations launched the Convention on the Rights of the Child [8]. Through participation, children can develop an appreciation of democracy, becoming empowered and playing a vital role in their own development and that of their communities [8, 9]. Studies have shown that involvement of children and adolescents in planning and implementing health promotion measures has improved the applicability and sustainability of such measures and led to better competencies and health-related outcomes [10, 11].

In Norway, children’s right to participate has been included in the Norwegian Planning and Building Act [12] and the Norwegian Public Health Act [13]. Since 2019, Norwegian municipalities have been obliged by law to establish a young people’s parliament or youth council [14]. Under certain circumstances, the municipal councils are also obliged to collect and assess suggestions from the population and, in general, ensure broad and open participation from various groups in decision-making processes.

Despite the growing body of evidence on the importance of involving adolescents in community measures, there is a lack of studies investigating factors influencing the participation of young people. Some studies have investigated what makes participation successful [15, 16], and some have looked into challenges related to the involvement of citizens [17, 18]. Furthermore, the adult actors facilitating co-creation processes have been shown to be important for how the involvement becomes realised. For example, buy-in from adult stakeholders was found to be critical for the implementation of youth–adult partnerships [19], but such processes also require support, resources and training of stakeholders to succeed [19, 20]. However, several of these studies investigate co-creation processes in contexts other than public health, such as in democratic governance processes in general or governance of environmental issues, or they do not involve adolescents. Thus, there is a need to explore co-creation processes with adolescents within the field of public health. Our research question was the following:

What do municipal actors consider as inhibiting and promoting adolescents’ involvement, at ages 10 to 19, in public health measures in municipalities?

In this study, ‘municipal actors’ refers to individuals who are central in involving adolescents. These include project leaders and project group members linked to the National Programme for Public Health Work in Municipalities (2017–2027) in Norway, which focuses on systematic and long-term public health work to promote children’s and adolescents’ mental health and wellbeing [21]. The term ‘municipal actors’ also refers to teachers, because many municipalities involved in the programme organise involvement processes through schools, making teachers a key resource. Municipalities participating in the programme develop public health measures based on local needs assessment and the involvement of the local population. A programme sub-goal is to establish routines for the participation and involvement of children and adolescents in health promotion measures [21]. Each municipality has an interdisciplinary project group developing and implementing the measures in collaboration with adolescents. Adolescents’ experience of the co-creation process is explored in a parallel study.

## Methods

### Sample and procedures

Five municipalities were included based on a set of criteria. They all had to be part of the National Programme for Public Health Work in Municipalities in a county in central Norway, they were developing a public health measure targeting adolescents, and they had a defined plan for user involvement. We also ensured that the included municipalities represented variations in population size and location in rural and urban districts. This was done to explore municipalities with a variation in available resources and number of adolescents. See Table I for more information about municipalities, their chosen measure, involvement of adolescents, and interview participants.

**Table I.** Population, measure, methods and arenas for adolescent involvement and interview participants.

Municipality	As of 1 January 2022^a^	Measure	Methods and arenas for adolescent involvement	Interview participants
Municipality 1	2000	Establishment of a leisure club	The Youth Council was the designated project group Pupil Council (occasionally consulted) Questback survey was administered to 5th to 10th graders Pupils in lower secondary school were involved in creative work during classes (making cardboard models and videos)	Project leader (drug and crime prevention coordinator)
Municipality 2	6000	Schoolyard renovation	Pupil Council (consulted by the project leader) Pupils in lower secondary school were involved in creative activities during classes (carpentry, painting, etc.) Pupils in primary and lower secondary school took part in a drawing competition (‘My Dream Schoolyard’) Pupils were consulted by an architect and researchers (interviews and GPS registration of schoolyard activities) Adolescents and adult stakeholders took part in an ‘activity night’ with image scraping	Group 1: project leader (primary school principal), two project group members (teacher and advisor in spatial and community planning) Group 2: six teachers
Municipality 3	15,000	Parent supporting measure	Questback survey was administered to pupils in lower and upper secondary school Pupils in lower secondary school were interviewed by researchers Two groups of adolescents were involved in writing a song reflecting results from the Questback survey and attending music and studio courses	Project leader (public health coordinator), two project group members (teacher and head of a youth centre)
Municipality 4	2000	Establishment of a youth club	Pupil Council members were represented in the project group 8th graders were involved in creative activities during classes (cardboard modelling, image scraping, room sketching, etc.) Prioritising challenges with pupils in lower secondary school (using the digital tool Mentimeter) Brainstorming with pupils in lower secondary school (using the digital tool Padlet)	Project leader (public health coordinator)
Municipality 5	6000	Schoolyard renovation	5th to 10th graders were involved in an activity night/brainstorming with adult stakeholders (group work aimed at prioritising wishes and needs for a schoolyard) 5th to 10th graders were consulted by the project leader during school hours Pupil Council (occasionally consulted by the project leader)	Project leader (public health coordinator)

a Numbers retrieved from Statistics Norway [22], rounded off to nearest 1000.

A qualitative approach with individual and group interviews and participatory observation was chosen. We interviewed project leaders and members of the project groups in five municipalities as they were central actors in involving adolescents. The interview participants consisted of various categories of people: public health coordinators (*n*=3), teachers (*n*=2), drug and crime prevention coordinator (*n*=1), primary school principal (*n*=1), advisor in spatial and community planning (*n*=1), and a head of a youth centre (*n*=1). These were asked to suggest others who played key roles. In one municipality, a group of teachers (*n*=6) was defined as central actors and was interviewed in a separate group. Altogether, 15 persons were interviewed.

Originally, we wanted to conduct group interviews to elicit discussion around interview participants’ experiences on the subject. However, due to COVID-19 pandemic precautions and how the project work in each municipality was organised, we ended up conducting a mix of individual (*n*=3) and group (*n*=3) interviews. Project group sizes varied from municipality to municipality – from six persons to only one person in one municipality. The group interviews included three to six participants. Individual interviews were conducted digitally due to COVID-19 restrictions, while group interviews were done in person. All interviews lasted from 45 to 80 minutes. The first author conducted individual interviews, and a fellow researcher accompanied the first author in all group interviews.

We used a semi-structured interview guide addressing three main topics: (a) the municipality’s public health measure and the project process linked to the national programme; (b) participation and involvement of adolescents in general; and (c) how the municipality involved adolescents in their project. Examples of questions under each topic were ‘Can you describe how you have been working with the public health measure in your municipality?’, ‘In your opinion, why should children and young people be involved in measures targeting them?’ and ‘Can you describe something you have experienced as positive/negative in the process of involving adolescents and why?’. The interview guide consisted of 12 questions. Interviews were audio-recorded and transcribed verbatim by the first author (a total of 67 transcribed pages in an 11-point font).

Prior to the interviews, participatory observation of project activities in which adolescents were involved was conducted in two of the municipalities. This was done to gain contextual insights, deepen the understanding of what the interviewees said in the interviews, and inform the interview guide. The researcher took field notes immediately after attending the project activities. Due to COVID-19 restrictions, observation was not conducted in all municipalities. Data were collected during 2021.

### Analysis

A data-driven thematic analysis inspired by Braun and Clarke [23] was conducted by the first and third authors by following the prescribed steps (see Table II for analysis process). The analysis drew heavily on the interview transcripts with data from observations to understand further what the interviewees were talking about. All the interviews contributed to the results.

**Table II.** Analysis process, adapted from Braun and Clarke [23].

Read transcripts to obtain an overview
Conduct initial coding of each interview
Search for patterns across transcripts and form themes
Review themes, going back and forth between codes and themes. In this phase, we merged some of the candidate themes
Define and refine themes, clarify the focus and name themes
Write the manuscript by telling the story of the data supported by illustrating data extracts. As analysis is an ongoing, organic process, we went back and forth between the phases, coding and re-coding several times. In addition, we reread the dataset to ensure that all pertinent information was coded and to avoid missing the meaning of the dataset as a whole

### Reflexivity

The authors work in the field of public health. While the first author is a young researcher with limited research experience, the second and third authors are experienced researchers. In line with Braun and Clarke’s methodology on reflexive thematic analysis, we acknowledge that our previous experiences, assumptions, and beliefs will influence the research process and our findings. Rather than striving for objectivity, we try to be as transparent as possible regarding our methodological choices and assumptions so that the reader can gauge our choices and decide the findings’ transferability [23].

### Ethics

The Norwegian Agency for Shared Services in Education and Research assessed the study and found it to be in accordance with national ethical standards for research (protocol code 451348). The study was also submitted for consideration to the Regional Committee for Medical and Health Research Ethics Central Norway (REC). However, it was not deemed necessary for approval because the study was not classified as health research (application number 334116). All participants volunteered and gave their written informed consent. Confidentiality was emphasised. The research was conducted according to the guidelines of the Declaration of Helsinki.

## Results

The municipal actors reported factors they experienced as influencing the involvement of adolescents. In the analysis, we developed four themes, including both inhibitors and promoters for adolescent involvement: (a) timeframe challenges in adolescent involvement; (b) lack of necessary knowledge and awareness among adolescents; (c) limited competencies and resources in the project groups; and (d) facilitators’ attitudes on and perceptions of adolescent involvement. Each theme could serve as both a promoter of and inhibitor for involvement. For instance, limited resources in the project groups served as an inhibitor, and the opposite – an abundance of resources – was highlighted as a promoter. Likewise, certain attitudes on and perceptions of involvement among facilitators were viewed as promoting involvement, while other attitudes seemed to inhibit involvement. In the following, we explain these four themes further.

### Timeframe challenges in adolescent involvement

The interviewees highlighted a long project duration as a key inhibitor for involving adolescents. The projects lasted years, as it took time to plan and implement the measures. Also, lengthy municipal processes and delays related to COVID-19 restrictions delayed the process further. According to interviewees, long-term projects were problematic because it was viewed as crucial that the involved adolescents could see the results to get a clear sense that their efforts had produced actual outcomes. In one municipality, where the measure involved renovation of the outdoor school area and building a wall for playing ball, one teacher said:

One of the problems [. . .] is that it takes time; that is, the time span from when they start to ask and wish [. . .] for that wall by the soccer pit. They have probably wanted it – maybe I exaggerate – for almost 10 years. Sometimes I’m unsure if the kids will say they have been involved in the decision of getting that wall if you ask them.

Most project periods extended beyond a school year, and interviewees said this led to difficulties in maintaining continuity in the involvement. Some believed it was harder to engage the older group of adolescents, as they would be off to upper secondary school before the completion of the undertaking. On the other hand, the younger adolescents often showed signs of impatience, wanting things to happen right away. In general, it was seen as challenging to keep the various groups motivated over longer periods.

Conversely, the interviewees reported that the adolescents needed time and space when involved in decision-making to familiarise themselves with the project group members, the details of the projects, and, as one municipal actor explained, ‘what the former youth council had done before them’. Interviewees described themselves as ‘bridge builders’, taking care of things until new youth council members could get up to speed on their responsibilities.

While the steady shift of pupils entering and leaving the projects was seen as challenging, some saw positive outcomes. They emphasised that new pupils were ‘bubbling over with ideas’ and that the projects benefitted from a constant stream of new initiatives. Interviewees also talked about strategies they had applied to work around problems caused by the lengthy timeframes. One was to include younger adolescents as this would improve their chances of seeing end results before transferring to upper secondary school. Other strategies involved designing less extensive projects or implementing measures in steps so those involved could see and enjoy parts of the results. Project leaders also tried to celebrate the smaller wins along the way:

I thought it was rather nice to have a small marking now. An opening. Because if we had waited until the end of the project, many of the pupils would have been gone.

### Lack of necessary knowledge and awareness among adolescents

The interviewees pointed to how the younger groups had limited knowledge and experience in participating in previous projects; thus, they were not used to collaborating on behalf of their age group. However, the pupil council representatives were more accustomed to collecting ideas and suggestions from their peers and conveying information to them about the ongoing projects. Although municipal actors suspected some information was lost along the way, they found it hard to instruct the adolescents and to keep track of the quality of the information flow.

It is difficult to tell the pupil council that they should inform their classes. At least when it comes to 5th and 6th graders, a lot of information will get lost. And a lot of meaning in the information also gets lost.

Others were not too worried about this and instead accepted that some information got lost on its way to the larger target group. They considered the process had valuable learning outcomes for the adolescents in any case: learning to collaborate with others, raise their voices, and take part in democratic processes for the local community. Many stressed the importance of adjusting involvement strategies and the information provided to suit the specific age groups and their level of knowledge and experience better. They also said they spent a lot of time and effort on this. One stated, *‘*If you are going to meet them to really listen to them, you somehow need to adapt to the level or how they want it served’.

Various methods and tools were used for involving adolescents, but which adolescents and how they were going to be involved had most often already been decided by the adult members of the project groups. One project leader did, however, emphasise the importance of letting the adolescents themselves choose how and how much they wanted to be involved and which tools or strategies should be used to ensure their engagement. In the project of that specific municipality, digital tools such as Padlet, which is a web application similar to a digital corkboard, were used, as was the web-based polling tool Mentimeter. The project leader said this was to ensure broad involvement, including from those wanting to participate anonymously. She had experienced that not all adolescents were comfortable sharing their wishes and opinions in front of others.

Despite such efforts, the interviewees expressed worries that the adolescents would not understand what involvement was and what it should entail. Often, they had to remind them that they were being involved. Many adolescents, they said, seemed to believe that involvement meant they could decide everything. In addition, the adolescents would not always understand what needed to be considered when planning measures, such as economic and contextual restraints. It was important to clarify expectations to avoid disappointment.

When creating involvement processes, you must have a way of orienting the reality of what is possible to avoid too high expectations. That we are starting something huge, and then they are like, ‘oh, so there’s just a ball wall there’.

The municipal actors highlighted and problematised the fine line between maintaining motivation and enthusiasm among the adolescents and instilling a realistic vision of what was achievable.

The balance between not killing the motivation and the eagerness to suggest and see things happen. There is never anything but good ideas and good intentions, but it’s not certain that everything is feasible. And then it’s bad if they get the impression that this is feasible, and then they push on and face reality later than they could have done. I think it’s better that it’s stopped early, so they can let go of the thought and rather move on with something else. Because there’s no doubt that they want to contribute. They want things to be different, that things will be better.

### Limited competencies and resources in the project groups

Lack of competence and resources among the actors in the project groups was highlighted as a factor that hindered involvement. Several interviewees said they wanted to involve more adolescents but had to limit the number due to a shortage of resources. In addition, some interviewees said they lacked competence when it came to planning and implementing the measures or involving adolescents. In one municipality, they hired an architect who engaged the adolescents:

He managed to contribute, to have professionalism when he talked to the children. This enabled him to bring out more in the conversations with the children than I would have managed. I think that was a great way to get out the most potential from the little children.

The project leaders found it challenging to balance both their roles and time because the project often came on top of other work, and they did not receive additional compensation to complete the projects and ensure involvement.

Limited resources forced them to be creative in finding ways to involve the young. Existing structures and resources such as pupil councils, youth councils, existing projects, and various school courses were used to facilitate involvement in planning and implementation. In one municipality, hiring a milieu therapist to follow up with pupils with tailored educational needs had been a coincidental but crucial factor for succeeding in involving this specific group and completing the project. He coordinated and engaged the pupils in a practical manner; that is, doing carpentry and painting as part of renovating the schoolyard.

Even though involving adolescents was time-consuming and demanding, the interviewees found it important to spend extra time on doing so.

The challenge is that everything takes a longer time. It is much faster to do the preparations for them and just present it to them. And I think that it is often done in municipalities in Norway. So, I think you have to take that time and allow them to do it. If not, there will be many failed measures that are not necessarily what they are most interested in. So, I think it is good economy to give them time.

### Facilitators’ attitudes on and perceptions of adolescent involvement

The fourth factor affecting the involvement process was attitudes on and perceptions of involvement among those facilitating the involvement processes. Several interviewees said that attitudes and perceptions among involved adults influenced involvement; that is, how adolescents got involved and the level of involvement achieved. Some seemed most concerned with the end result of the project, while others emphasised the involvement process itself and the possible outcomes of being involved.

It’s easy to tick off the box for adolescent involvement. I think it’s a little too easily done. You can get away with it in many ways. What I think is important, and what I have tried to do in the project, is to let them have experiences along the way of reaching the goal and let them have their own voice without dictating what they should think. That has not necessarily been managed by the adults. I think the result can be improved by letting them work on things, not just being presented with something that is already completed, and then they get to be involved. Rather, they should be allowed to work and be involved in the processes from an early stage.

All interviewees found involvement important and related it to better-tailored, sustainable measures and skills development among adolescents. They also believed it could strengthen the school and community environment and that having positive experiences of involvement could motivate the adolescents for later community engagement and increase their participation in elections.

If you think about it, this is basically a good practice for later participation in elections. In the future, we will not have that many election absentees because we try to make them understand how important it is to participate.

When we asked why involvement is important, one interviewee said:

I think it means a lot in the general lives of children and adolescents that they are allowed to take part in important decisions and start believing that they are worth more than just what they accomplish. I see a big difference in the pupil council as well – a big difference from when they start in the pupil council and after they have been there a few years. It’s easier for them to come up with well-founded suggestions, and they improve their ability to listen to each other and understand the dynamics of expressing opinions. It is a very useful education towards growing up, at least.

In addition, interviewees interpreted their own role and the issue of involvement differently. Some saw themselves as those who make the decisions while merely consulting adolescents, others as facilitators and advisors, giving centre stage to the adolescents. One project leader had excluded adult members from some project meetings because she found them too dominating, steering the adolescents too much. She explained that she did it to give the adolescents time and space to think more freely, as she wanted them to feel ownership of the project. She seemed more concerned with the involvement process than the end result of the measure.

## Discussion

The interviewees experienced several factors affecting the involvement of adolescents, and these factors are closely linked. Moreover, our findings show that municipal actors worked hard to counteract the inhibiting factors by being creative and flexible, using several means and strategies. According to the interviewees, involving adolescents presented challenges related to their age. They experienced that the young people had limited decision-making skills and, in addition, needed time to get properly involved in the projects. Municipal actors, therefore, had to provide adolescents with the right tools and knowledge to participate beyond mere tokenism. Previous studies indicate that citizens often have little understanding of the goals and constraints of other actors and suffer from information deficits and asymmetries [17, 24]. Hence, educating adolescents is an integral part of a successful involvement process. The municipal actors in our study said they contributed to the adolescents’ democratic education and thus seemed to play an important role in providing decision-making and community development experiences. This is especially important, as there is a growing concern across Europe about the extent to which young people participate in society [25]. In line with the positive development approach for children and adolescents [26], participation in community development processes can be empowering, providing the individual with competencies or coping strategies and new tools to improve their lives.

Furthermore, our findings indicate that municipal actors lacked the competence and resources to engage adolescents and that there were few or no systems or structures supporting them in their efforts to involve the young – despite national guidelines and policies emphasising the critical importance of obtaining citizens’ involvement. Thus, the organisation of involvement processes relied heavily on the specific engagement, inventiveness, skills and networks of the individuals in the project group. This also meant that the project members’ attitudes on and perceptions of involvement and engagement of citizens influenced the involvement processes and how these turned out.

The attitudes or frames of mind of the municipal actors could be arranged along a spectrum from those most concerned with the end results and not so much the process of involving the young to those more eager to get the involvement processes right and not being too concerned about the measure itself or the result. Similar findings have been reported in other studies in which the personal characteristics of facilitators of involvement were found to be crucial to the involvement and realisation of the process. A study conducted in the USA [27] reported that finding and keeping capable facilitators was especially significant. The literature also indicates that transformational leadership, in which leaders take the role of visionaries, facilitates bottom-up engagement processes [28].

Another factor influencing the involvement was project timeframes. Lengthy projects spoiled the adolescents’ opportunity to enjoy the outcome or see the results of their efforts, and made the interviewees question whether the adolescents experienced any influence. Municipal actors linked the long project durations to inefficient bureaucratic processes in the municipalities, which made it challenging to keep the adolescents motivated. Previous research has shown that bureaucratic structures strongly influence citizen engagement, promoting or inhibiting involvement [19, 20, 24]. This is in alignment with our findings.

Even though the National Programme for Public Health Work in Municipalities emphasises the importance of involving adolescents and puts pressure on municipalities to comply with the goal of involving citizens and making them co-creators, our findings indicate that it was ultimately left to the individual municipal actors how this should be done in practice. This is in line with a Norwegian study in 2015, which found that the responsibility for involving citizens was left to public health coordinators [29]. In the municipalities investigated in our study, this still seems to be the case now.

### Methodological reflections

Although we aspired to recruit municipalities varying in population size and rurality/urbanity, most municipalities were rural and sparsely populated. This must be taken into account when considering the transferability of the findings. The municipalities had reached different stages in the process of co-creating measures. This may have influenced the experiences the municipal actors had and which aspects they emphasised. In addition, the study was carried out during the COVID-19 pandemic, which may have affected our findings.

A strength of the study is the triangulation of data sources. While we originally wanted to conduct only group interviews because we sought to facilitate discussion, we experienced that the individual interviews offered more depth [30]. The use of observations enabled us to understand better what interviewees talked about during interviews and helped us ask better questions. A limitation of the study is that observation was not conducted in all municipalities due to COVID-19 restrictions. However, emphasis was placed on the interview data in the analysis and reporting of findings. Also, the current study does not include the perspectives of involved adolescents, which is a limitation. Further research is needed to explore adolescents’ experience of co-creation processes.

## Conclusions

A key finding in our study is that those facilitating co-creation processes with adolescents experience that it takes time to involve the young and build their skills in democratic decision-making and participation. However, the results also show that overly long timeframes are not advisable either as municipal actors report it as crucial that adolescents involved can enjoy the results of their efforts before they have ‘outgrown’ the outcomes or leave school to enter high school.

The study also indicates that the methods for involvement should be varied, facilitating different ways of being involved. Even though our study did not specifically address issues related to the participation of less advantaged groups, varying methods for involvement will most likely increase the possibility of allowing for broader involvement among adolescents, including the most disadvantaged. Finally, the results showed that the municipalities participating in the study developed and experimented with methods and means for involving the young, always seeking to find ways to engage and involve the target groups. However, involving them depended strongly on the understanding and efforts of the municipal project group. It also became evident that co-creation represents new ways of working and collaborating in the municipalities, and that finding good practices for realising co-creation takes time and effort from all stakeholders involved.

Our findings suggest that further work needs to be done to ensure the involvement of adolescent citizens in municipalities despite the increasing volume of policies and legislation emphasising the importance of involving citizens. To do this, municipal actors must be provided with the necessary resources, competencies, and structures to facilitate involvement so that the involvement processes and their outcomes are not dependent on individual actors alone.

In addition to considering the factors influencing involvement identified in our study, further research is required to explore how adolescents with different socioeconomic backgrounds can be engaged in public health work. This is important in avoiding the unintended effects of increasing social inequalities in health. Children and adolescents are usually underrepresented in political decision-making processes. Engaging them broadly in such processes is rooted in the belief that to solve complex problems for future generations, we cannot just work for them; we need to work with them.
